# Algorithm for Video Summarization of Bronchoscopy Procedures

**DOI:** 10.1186/1475-925X-10-110

**Published:** 2011-12-20

**Authors:** Mikołaj I Leszczuk, Mariusz Duplaga

**Affiliations:** 1 Department of Telecommunications, AGH University of Science and Technology, Krakow, PL 30059 Poland; 2 Jagiellonian University Medical College, Krakow, PL 31-531 Poland

## Abstract

**Background:**

The duration of bronchoscopy examinations varies considerably depending on the diagnostic and therapeutic procedures used. It can last more than 20 minutes if a complex diagnostic work-up is included. With wide access to videobronchoscopy, the whole procedure can be recorded as a video sequence. Common practice relies on an active attitude of the bronchoscopist who initiates the recording process and usually chooses to archive only selected views and sequences. However, it may be important to record the full bronchoscopy procedure as documentation when liability issues are at stake. Furthermore, an automatic recording of the whole procedure enables the bronchoscopist to focus solely on the performed procedures. Video recordings registered during bronchoscopies include a considerable number of frames of poor quality due to blurry or unfocused images. It seems that such frames are unavoidable due to the relatively tight endobronchial space, rapid movements of the respiratory tract due to breathing or coughing, and secretions which occur commonly in the bronchi, especially in patients suffering from pulmonary disorders.

**Methods:**

The use of recorded bronchoscopy video sequences for diagnostic, reference and educational purposes could be considerably extended with efficient, flexible summarization algorithms. Thus, the authors developed a prototype system to create shortcuts (called summaries or abstracts) of bronchoscopy video recordings. Such a system, based on models described in previously published papers, employs image analysis methods to exclude frames or sequences of limited diagnostic or education value.

**Results:**

The algorithm for the selection or exclusion of specific frames or shots from video sequences recorded during bronchoscopy procedures is based on several criteria, including automatic detection of "non-informative", frames showing the branching of the airways and frames including pathological lesions.

**Conclusions:**

The paper focuses on the challenge of generating summaries of bronchoscopy video recordings.

## Background

Bronchoscopy examinations last from several to 20 or more minutes. Depending on the approach, video recordings of the procedure vary considerably in length. In some bronchoscopy suites only meaningful shots and sequences are recorded for documentation. However, due to the growing pressure of quality assurance and liability problems, it may be preferred to record the entire procedure. This means that images of varying quality are recorded. It is a common finding in videos recorded during endoscopic procedures that the meaningful part of the recording includes frames which are blurred or carry no significant information. Frames which are of poor quality because of blurring or for other reasons have been named by some authors as "non-informative" frames. According to [1], the percentage of such frames in video recordings of colonoscopies can reach as high as 30%. The same applies to video recordings of bronchoscopy procedures due to the high mobility of the trachea and bronchial tree, and the discharge of fluids and excretions which can adhere to the lens located in the tip of the bronchofiberoscope. Frames blurred due to motion, secretions covering the bronchofiberoscope lens or due to viewing an area outside the focus range make up a considerable part of video recordings of bronchoscopic procedures. Such recordings are of limited use for documentation or for educational purposes. On the other hand, physicians performing endoscopic procedures have limited time for processing the recordings. Manual exclusion of non-informative frames and selection of key sequences are time-consuming activities. It is also of no value to students or physicians in training to view resources of limited informative value. This brings a demand for automatic or at least semi-automatic methods of creating summaries of bronchoscopy video recordings.

Efficient summarization of video recordings of bronchoscopic procedures is also highly relevant for the development of medical video libraries for bronchoscopists. This strategy should cover the issue of exclusion of non-informative frames, as well as inclusion of those sequences and frames which are crucial for documenting the normal appearance of the endobronchial environment or presenting pathological lesions revealed during examination. Thus, automatically generated summaries could be of considerable value in supporting long-term archiving of video resources or efficient access to medical video library resources. The second option may be enhanced with ad-hoc adjustment of the summarization criteria.

Selecting the most relevant shots from the whole video sequence is one of the most essential problems in bronchoscopy video summarization. The perception of the problem is not fully agreed upon among medical specialists. A mechanism to facilitate this process should include an algorithm enabling the automatic analysis of the shots forming the video and including those carrying essential information about the examination.

As mentioned above, the process covers a number of aspects related to the analysis of the content of the video sequence, allowing for identification of meaningful shots and scenes [2] and other parts of a coherent theme [3], or for presentation in a hierarchical form [4], [5], [6], [7]. There may be hierarchical relationships between parts of one sequence of the video, as well as between different video sequences [8].

Unfortunately, current medical digital video libraries do not include summary subsystems. The challenge of summarizing video sequences with medical content has rarely been studied by other authors [9], [10], [11]. Vilarino et al. [11] have presented an interesting approach, although it cannot really be applied to bronchoscopy video recording since it is in a different medical domain. Choupo et al. presented a method of clustering large image databases [12]. However, their paper considers general multimedia content and so it has no strict relation to the specific type of images considered here. The approach presented by Arevalillo-Herráez et al. [13], based on involving end-users in content-based image retrieval, which was applied to summarizing generic digital video libraries, does not depend on the modality of medical images. Due to specific features of bronchoscopy videos, a method tailored strictly to their content needed to be created, based only partly on related approaches, including the abovementioned ones.

The authors assumed that the summary must be considerably shorter (ten or more times) than the whole video sequence. However, the final rate of the initial sequence and summary lengths should depend on the aim of the summary preparation. Long-term archiving could require shorter summaries in comparison to short-term storage or presentations of the procedures for educational purposes. The general approach resulting from the authors' experience and consultations with professional film editors assumed that the volume of the summary should be reduced around twelve times [14] when compared to the original video, which means that one minute of the original video sequence should be illustrated by five seconds of the summary. At the same time an approach of creating summaries with a volume shorter than 30 seconds has not been adopted [14]. Nevertheless, the final degree of shortening should be based on user preferences and the projected usage of the summaries.

**Figure 1:**
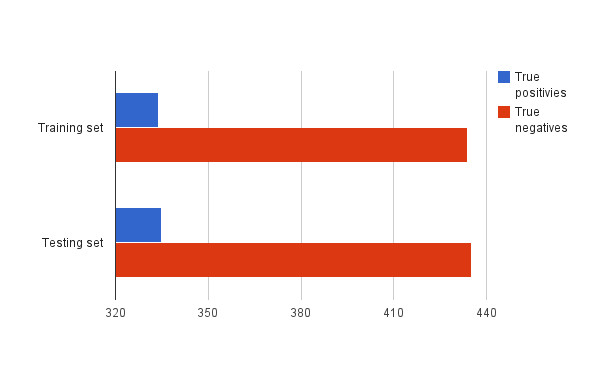
**The ground truth**.

This work is an extension of the earlier book chapter [15]. New significant contributions have been added into the "Methods" section. These are: information on details on how the evaluation has been performed, Figure 1 (related to the above-mentioned information), more discussion of sensitivity and specificity of the achieved results as well as distinction between state-of-the-art methods provided in the references and the proposed ones. Furthermore, new contribution on the scenario of the system usage in clinical environment has been added into the "Conclusions" section.

The authors developed a model of a prototype system for generating summaries of bronchoscopy video recordings. The model assumes an application of image analysis methods for excluding frames without informative value and selecting those which could have added value for diagnostic or educational purposes. It originates from previous research activities conducted within the BRONCHOVID project focused on the development of a computer system supporting bronchoscopy laboratories [14], [15], [16], [17], [18] and [19]. Related tributary models have been discussed in more detail in the "Methods" section.

The paper is structured as follows: the "Methods" section presents the algorithm developed to summarize bronchoscopy videos; the "Results and Discussion" section presents the implementation of the algorithms; and the "Conclusions" section concludes the paper and gives an insight into further development.

## Methods

The algorithm for the selection or exclusion of specific frames or shots from video sequences recorded during bronchoscopy procedures is based on several criteria. The main effect of this algorithm will be the inclusion of shots consisting of frames tagged with inclusion criteria and the exclusion of frames tagged with exclusion criteria.

A primary criterion for exclusion from the summary is related to information in a specific frame. The authors assumed that "non-informative" frames may be important for bronchoscopic assessment only in relatively rare cases. An overall poor quality of the whole recorded video due to independent conditions, e.g. profuse bleeding or the presence of specific excretions in the endobronchial space, may be an example of such a situation. The following criteria were considered for inclusion:

• markers (manually introduced) from medical treatment,

• frames annotated by the physician performing the bronchoscopy for reference purposes,

• frames showing the branching of the airways (ramifications),

• frames including pathological lesions detected automatically with the available algorithms of image recognition, complemented with representations of intermediate frames of the shots, and

• representative sample of frames between branching points (the ratio of "informative" frames included in the summary should be adjusted according to the purpose of the summary generation).

Methods which enable the application of these criteria are described below.

### "Non-informative" frames

**Figure 2:**
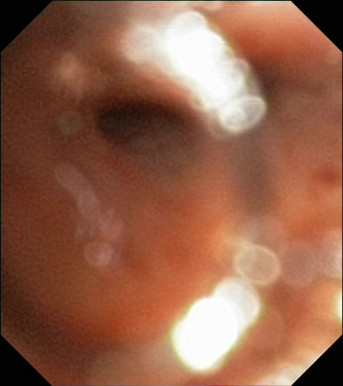
**Example of an "non-informative" frame**.

**Figure 3:**
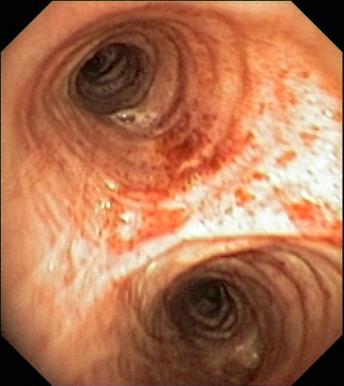
**Example of an "informative" frame**.

Video recordings of endoscopic procedures performed within airways include frames of both adequate and inadequate quality for assessment by an endoscopist. Frames of inadequate quality are called by some authors blurred or "non-informative" (see Figure 2 and Figure 3). The fraction of "non-informative" frames within a video recording of bronchofiberoscopy may be considerable and it varies from case to case.

Methods based on edge detection, HSV (Hue, Saturation, and Value) histograms with neural networks, MPEG-7 descriptors, and DCT (Discrete Cosine Transform) have been developed and tested. From all these methods, the authors selected the algorithm based on DCT due its highest sensitivity, specificity and F-measure of identification of "non-informative" frames within test sets prepared by endoscopists. This algorithm of detection of "non-informative" frames is based on the fact that images which do not contain numerous edges (as in the case of most types of "non-informative" frames) are easier to compress using algorithms based on DCT, such as used in the JPEG algorithm. Such frames reveal considerably narrower spectra in the frequency domain than frames which contain numerous edges. This feature of DCT allows it to be used as a simple yet effective edge detection algorithm. The DCT-based algorithm does not allow for localization of the edges in the image, but this is not necessary for the detection of "non-informative" frames.

**Figure 4:**
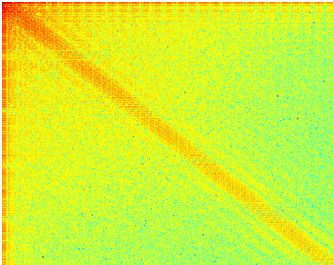
**Frequency spectrum of "non-informative" frames**.

**Figure 5:**
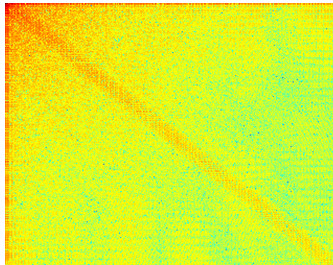
**Frequency spectrum of "informative" frames**.

Examples of spectra obtained from "non-informative" and "informative" frames were demonstrated in Figure 4 and Figure 5 respectively. The figure shows that in the "non-informative" frame the energy is condensed around the harmonics of the lower order, whereas in the "informative" frame the energy is more evenly distributed in the spectrum.

**Figure 6:**
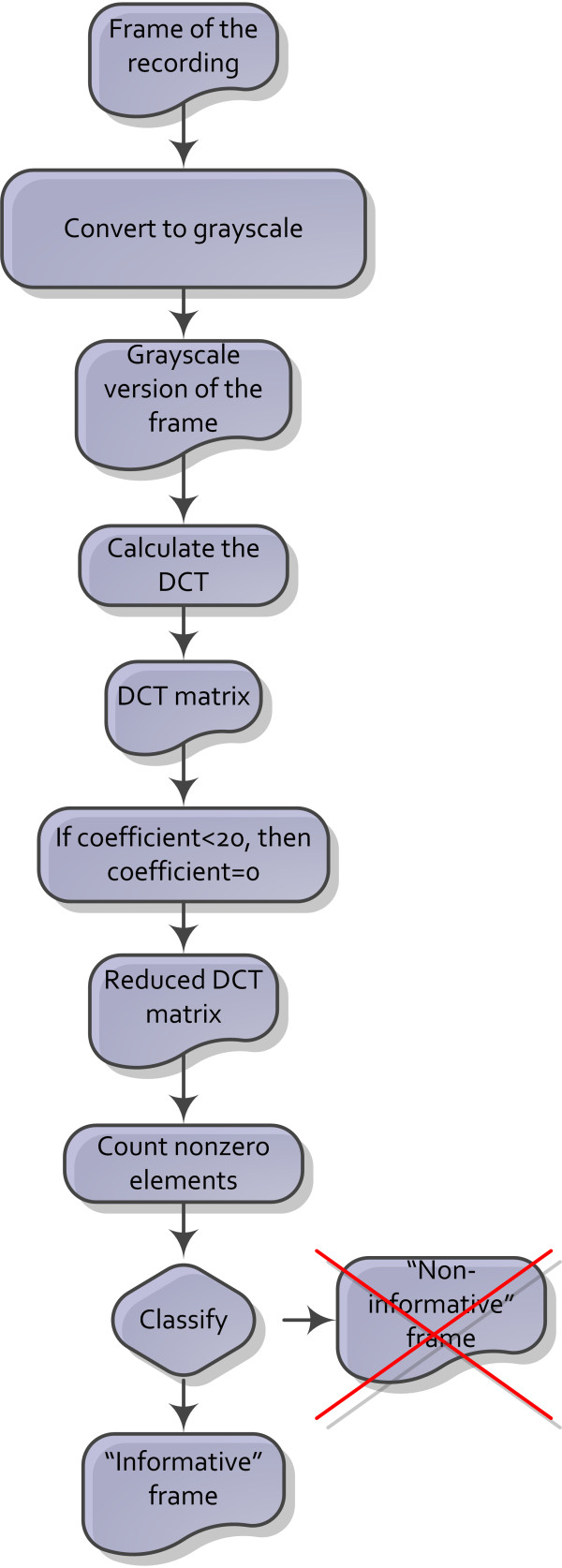
**The classification algorithm based on the Discrete Cosine Transform for "informative" and "non-informative" frames**.

The algorithm of DCT-based "non-informative" frame detection is presented in Figure 6. In the first step a frame from the bronchoscopy video recording is converted into a grey-scale image by discarding the hue and saturation while retaining the luminance. In the second step, the DCT coefficients of the image are computed. In the third step, the values of a magnitude lower than 20 are set to 0. The value of 20 was selected experimentally as it provides the best results in terms of precision and recall [19]; however, the algorithm can be easily re-tuned in case of custom sensibility demands. In the final step, non-zero elements of the DCT matrix are calculated and used for discrimination.

The evaluation has been performed on a set of 1538 frames, manually annotated as "non-informative" (669 images) and "informative" (869 images) by the experienced bronchoscopist. The set acted as a ground-truth. Then, the images were evenly (but randomly) split to: the training set and the testing set (see Figure 1). Four metrics have been used in order to analyse the performance of the algorithm: F-measure, sensitivity, specificity and accuracy. This set of metrics allows for a comprehensive assessment of performance. It is a standard set of metrics used to assess the performance of binary classification. The results of the performance assessment are presented in Table 1.

**Table 1:** Evaluation of the performance of the DCT blurring detection algorithm [18]

*Metric*	*Definition*	*Value*
F-measure	$F-measure=\frac{\frac{t_{p}}{t_{p}+f_{p}}\cdot \frac{t_{p}}{t_{p}+f_{n}}}{\frac{t_{p}}{t_{p}+f_{p}}+\frac{t_{p}}{t_{p}+f_{n}}}$	0.92

Sensitivity	$Sensitivity=\frac{t_{p}}{t_{p}+f_{n}}$	0.93

Specificity	$Specificity=\frac{t_{p}}{t_{p}+f_{p}}$	0.93

Accuracy	$Accuracy=\frac{t_{p}+t_{n}}{t_{p}+f_{p}+f_{n}+t_{n}}$	0.93

The algorithm and its evaluation have been described in detail in [18]. A related approach has been presented in [19] and in [15]. The references have presented four methods for detecting blurring in video frames recorded during bronchoscopic procedures. The results demonstrated in the references show that the most of the presented methods achieve F-measure, sensitivity, specificity and accuracy of at least 90% or higher. Most of the proposed algorithms should be tuned in terms of maximising their accuracy-related measures. This allows for turning the algorithms to be under- or over-sensitive, if the "golden mean" is not achievable (as in the case). The next research step anticipated preparation of the super-classifier, considering the presented algorithms as tributary classifiers. The plans also include inclusion of algorithms not described in the paper, as for example the algorithm presented in [20], based on hidden Markov model (HMM).

### Frames annotated by the physician performing the bronchoscopy for reference purposes

This is an obvious criterion for inclusion. Annotation of the frames with terms taken from the classification tree is an optional, offline operation which can be performed just after the completion of the procedure or after a greater delay when a specific type of pathological lesion or anatomical structure is searched for in a medical video library. The accuracy of such descriptions is considered to be very high. Furthermore, as an endoscopist can identify several types of pathological lesions or anatomical structures, the algorithm can distinguish between them and optionally attach different inclusion weights to them.

### Frames showing the branching of the airways

**Figure 7:**
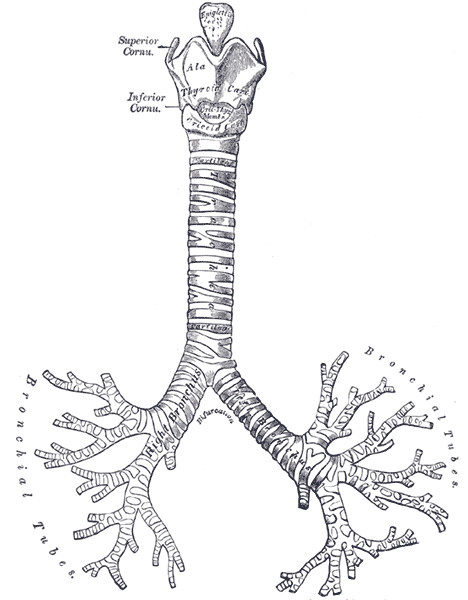
**The trachea-bronchial tree (source: public domain image, copyright expired)**.

Bronchoscopy is performed with the aim of assessing the lower part of the airways including the trachea and bronchi. The range of the examination depends on the structure of the bronchial tree and diameter of the fiberoscope used for the examination, although it usually does not exceed the level of sub-segmental bronchi. Lower airways form a tree-like structure with branching of the consecutive generations of this tree (Figure 7). The authors assumed that the most representative frames showing the division points of the trachea and bronchi should be treated as key points organizing the summarization process. The assessment of the ostia of next generation bronchial divisions is also an essential task in the visual assessment of endoluminal anatomy performed by bronchoscopists, as it can reveal changes resulting from pathological processes influencing the airway patency [21][22].

To ensure the inclusion of frames with airway branching points in the summary generated from the bronchoscopy video recording, an algorithm for automatic detection of such frames was developed by Mikrut et al. [23].

The algorithm is based on an observation that relatively large, dark (non-illuminated) areas seen within the lumen of the trachea or bronchi can be easily detected and counted. The presence of two or more such areas allows for the identification of a frame showing a view of a branching point of the airway tree. The algorithm first finds all dark objects, and then filters the results to pick out the objects surpassing the threshold value of the dimension. The basic concepts of image opening ^A^/closing ^B^, thresholding (by means of image complementing), labelling, and measuring the properties of image regions were successfully utilized.

### Frames showing pathological lesions detected automatically with available image recognition algorithms

Automatic detection of pathological lesions is another criterion for frame inclusion in summaries of video recordings of bronchoscopy examinations. The summarization system may utilize available algorithms of automatic detection and specifically those developed within the BRONCHOVID project including DWT (Discrete Wavelet Transform), MPEG-7 standard [24], and colour analysis. The number of automatically identified pathologies and the recognition accuracy are both still limited. Markings based on automatically discovered pathologies are accepted "as is", and further discussion about the way they appear and about their accuracy exceed the scope of this paper, as the algorithms have been described in detail in [25], [26], and [27]. The reference [27], even if indeed achieving better results, operated at slightly different data sets and for slightly different case, which is probably not directly possible for applications in our summarization algorithm. The results obtained in that work were on the set of frames after selection, and described in our recent article on the raw data set covering everything that has been recorded. During clinical trials both methods will be tested and selected for more effective practical action.

## Results and Discussion

At the time of submission of the paper, the module of video recording summarization was in the process of pilot implementation. The system uses a web-based GUI (Graphical User Interface).

**Figure 8:**
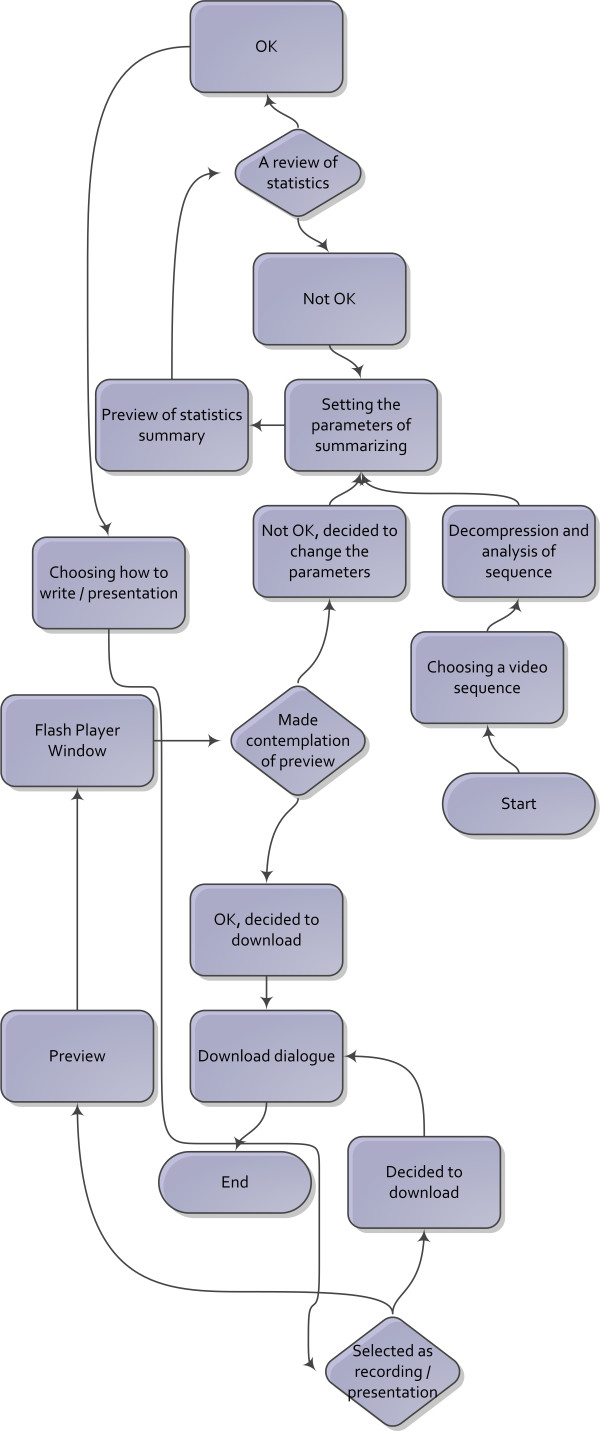
**The algorithm of GUI summarization**.

**Figure 9:**
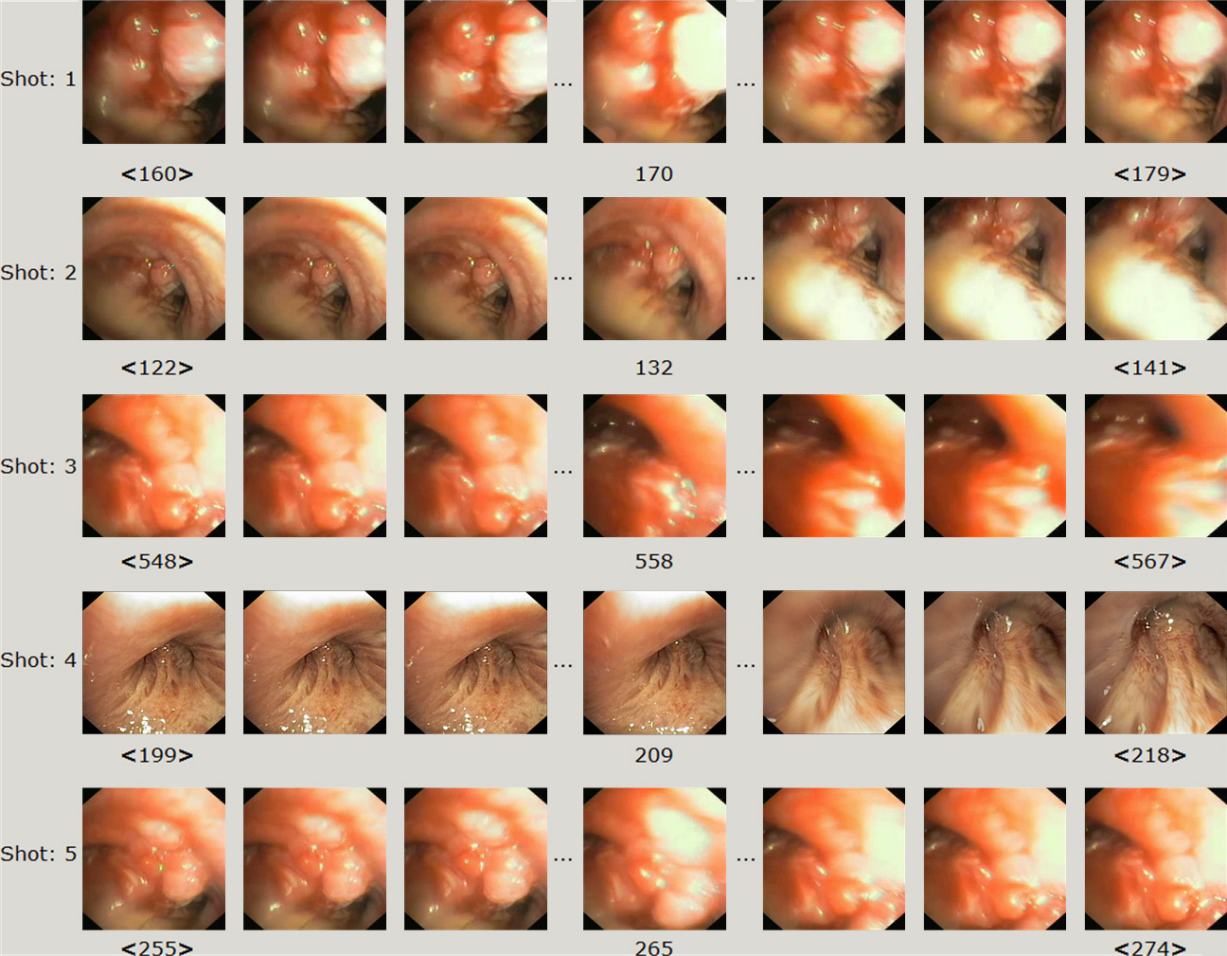
**Manual fine-tuning of automatically generated shots**.

**Figure 10:**
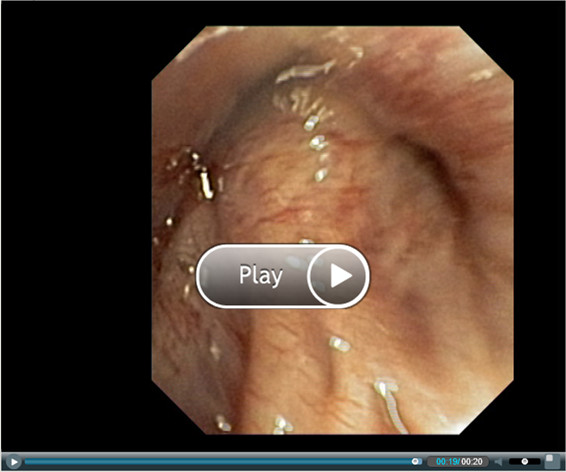
**Web-based playout of a generated summarized video**.

The algorithm of GUI summarization is shown in Figure 8. First, the user can select the video sequence for summarization. Partial decompression and analysis is carried out next. The next step anticipates the intervention of the person supervising the summarization algorithm and depends on the selection of summarization parameters. The process encompasses various options related to the definition of inclusion and exclusion criteria described earlier in this paper. Following this stage, the planned summary statistics can be reviewed and automatically generated shots can be manually fine-tuned (Figure 9). If the user is not satisfied the with summarization result, he is able to re-adjust the summarization settings. The summary may be previewed and downloaded. The summary can be previewed with Flash Player (Figure 10). After previewing the summary, the user can accept it and proceed to the download dialogue, or return to setting the summary parameters.

## Conclusions

The paper focuses on the challenge posed by generating summaries of bronchoscopy video recordings. The main rationale is improved efficiency of the archiving processes, as well as multi-purpose video resource browsing and presentation in educational and reference context in medical environments. The paper presented a summarization model. In later work we intend to publish the results of testing the system with clinical material. A prototype of the software described in the paper was developed by a broader project aiming at the platform supporting data management in bronchoscopy laboratories. The concept of the summarization algorithm currently implemented and tested in pilot settings was based on several assumptions resulting from previous experience in video summarization and the specific requirements of the medical community. The pilot implementation should provide feedback from end-users, enabling an assessment of the feasibility of the described approach.

The BRONCHOVID system was implemented in its pilot version in the Bronchoscopy Laboratory of the Department of Pulmonology, Jagiellonian University Medical College in Krakow. The Laboratory performs about 1,200 bronchoscopy procedures yearly. The bronchoscopy procedures are performed by 5 experienced pulmonary medicine specialists.

The BRONCHOVID system was used during the pilot period for:

- registration of the selected bronchoscopies,

- testing of the functionalities enabling annotation of the frames within registered videos,

- testing query function within the resources accumulated through pilot period,

- generation of summaries of registered bronchoscopy videos,

- download of selected shots from registered videos in commonly used formats for external usage, e.g. for educational purposes.

Currently, the scenario of the system usage starts from the system initiation just before the insertion of the bronchoscope to upper respiratory tract of the patient. The recording of the procedure is usually started by a nurse employed in the laboratory on request from the physician performing bronchoscopy. The whole procedure is recorded and the process is terminated after removing bronchoscope from the patient's respiratory system. The patient's data are entered after the procedure as the information on status of respiratory tract and findings during bronchoscopy are available then.

The anticipated system usage assumes recording of all procedures performed in the laboratory. Recorded bronchoscopy videos are planned to be kept in the full version for the period of 12 months. Afterwards, an automatic summary generation will be initiated and resulting video summaries will be kept for longer period. The videos demonstrating specific lesions or especially valuable in terms of educational and training impact will be kept in unabbreviated version for longer periods.

## Conflicting interests

The authors declare that they have no conflicting interests.

## Authors' contributions

Mikołaj Leszczuk contributed to "Methods" (partly) and "Results and Discussion". Mariusz Duplaga contributed to "Background", "Methods" (partly) and "Conclusions".

## Author information

**Mikołaj Leszczuk**, PhD, is an assistant professor at the Department of Telecommunications, AGH University of Science and Technology (AGH-UST), Krakow, Poland. He obtained his MSc in Electronics and Telecommunications in 2000 and PhD in Telecommunications in 2006, both from AGH-UST. He is currently lecturing in Digital Video Libraries, Information Technology, and Basics of Telecommunications. In 2000 he visited Universidad Carlos III de Madrid (Madrid, Spain) for a scientific scholarship. Between 1997-1999 he was employed by several Comarch holding companies as Manager of the Research and Development Department, President of the Management Board, and Manager of the Multimedia Technologies Department. He has participated actively as steering committee member or researcher in several national and European projects, including: INDECT, BRONCHOVID, GAMA, e-Health ERA, PRO-ACCESS, Krakow Centre of Telemedicine, CONTENT, E-NEXT, OASIS Archive, and BTI. His current activities are focused on e-Health, multimedia for security purposes, P2P, image/video processing (for general purposes as well as for medicine), development of digital video libraries (particularly on video summarization), indexing, compression, and streaming subsystems. He has been a chairman of the "e-Health in Common Europe" conferences sessions. He has been a member of the IEEE Society since 2000. He has served as an expert for the European Framework Programme and Polish State Foresight Programme, as well as a reviewer for several scientific conferences and journals.

**Mariusz Duplaga**, graduated from the Medical Faculty at the Jagiellonian University Medical College in Krakow in 1991, and completed his doctoral thesis in medicine in 1999, specializing in internal medicine and respiratory medicine. Between 1995-2003 he made working visits to several NHS centres in the UK; University College London, UK; SINTEF, Trondheim, Norway; Municipal Epidemiological Centre, Barcelona, Spain; University of Antwerp, Belgium; and CHRU in Lille, France. He is currently working as a senior researcher at the Institute of Public Health at the Jagiellonian University (leader of courses on e-Health/Telemedicine/e-Inclusion) and the Department of Pulmonology, Medical Faculty, Jagiellonian University. Since 2002 he has been a project manager at the Centre of Innovation, Technology Transfer and University Development, Jagiellonian University. He has been a site leader or research participant in several research projects on medicine and IT use in health and social care: AIANE (BIOMED II); Medical Data Management (USAID); Hospital Data Management (PHARE); Krakow Centre of Telemedicine (SCI-TECH II); BIOAIR (5FP), PRO-ACCESS (5FP); HEALTHWARE (6FP); e Health ERA (6FP); MATCH (6FP, DG INFSO-6FP); and MPOWER (6FP). He has participated as a project coordinator or researcher in several projects funded by the Polish Scientific Research Committee and the Ministry of Health in the areas of telemedical services, telemonitoring in severe asthma, and epidemiology of allergic diseases. Between 2000-2003 he took part in establishing the Krakow Centre of Telemedicine, and served as President of the Polish Association of Medical Internet. Between 2000-2002 he served as a delegate of the Polish Scientific Committee and the Ministry of Health to the EC Information Society Technologies Working Party on Health and Persons with Special Needs. In 2008 he served as an expert at the National Centre of Research and Development within the Ambient Assisted Living Programme. His main areas of research and development include: endoscopy, image processing, e-Health/telemedicine, e-inclusion, and support for persons with special needs, chronic care, telemonitoring, and computer-aided interventions.

## Endnotes

^1 ^The dilation of the erosion with various sizes of morphological structuring elements (image processing term)

^B ^The erosion of the dilation with various sizes of morphological structuring elements (image processing term)
